# A resting-state functional magnetic resonance imaging study of altered functional brain activity in cardiac arrest survivors with good neurological outcome

**DOI:** 10.3389/fneur.2023.1136197

**Published:** 2023-04-20

**Authors:** Qian Wu, Gan-Nan Wang, Hao Hu, Xu-Feng Chen, Xiao-Quan Xu, Jin-Song Zhang, Fei-Yun Wu

**Affiliations:** ^1^Department of Radiology, The First Affiliated Hospital of Nanjing Medical University, Nanjing, China; ^2^Department of Emergency, The First Affiliated Hospital of Nanjing Medical University, Nanjing, China

**Keywords:** cardiac arrest, cognitive impairment, resting-state functional magnetic resonance imaging, amplitude of low-frequency fluctuation, regional homogeneity

## Abstract

**Purpose:**

To investigate the spontaneous brain activity alterations in survivors of cardiac arrest (CA) with good neurological outcome using resting-state functional magnetic resonance imaging (rs-fMRI) with amplitude of low-frequency fluctuation (ALFF) and regional homogeneity (ReHo) methods.

**Materials and methods:**

Thirteen CA survivors with favorable neurological outcomes and 13 healthy controls (HCs) were recruited and underwent rs-fMRI scans. The ALFF and ReHo methods were applied to assess the regional intensity and synchronization of spontaneous brain activity. Correlation analyses were performed to explore the relationships between the mean ALFF and ReHo values in significant clusters and clinical parameters.

**Results:**

The survivors of CA showed significantly decreased ALFF values in the left postcentral gyrus and precentral gyrus and increased ALFF values in the left hippocampus and parahippocampal gyrus than HCs. Significantly decreased ReHo values were observed in the left inferior occipital gyrus and middle occipital gyrus in the patients. Mean ALFF values in the left hippocampus and parahippocampal gyrus were positively correlated with the time to return of spontaneous circulation (r = 0.794, *p* = 0.006) in the patient group.

**Conclusion:**

Functional activity alterations in the brain areas corresponding to known cognitive and physical impairments were observed in CA survivors with preserved neurological function. Our results could advance the understanding of the neurological mechanisms underlying the residual deficits in those patients.

## 1. Introduction

The survival rate of cardiac arrest (CA) has improved in recent years, however, CA is still one of the leading causes of death worldwide (1). Neuron damage can occur shortly after CA, leading to varying degrees of ischemic-anoxic brain injury (2). Although some CA survivors can achieve relatively good neurological outcomes and independent daily living (3, 4), these patients can still suffer from persistent cognitive impairments for a long time (5, 6). Cognitive deficits, including memory, attention, and executive dysfunctions, could reduce patients’ quality of life and cause social participation difficulties (7–9). A better understanding of the underlying neuropathological basis of the residual cognitive deficits could contribute to improvement in follow-up and subsequent therapy.

Several recent studies have shown that patients with CA possess structural and functional brain abnormalities (10–15). Koenig et al. (13) demonstrated decreased functional connectivity within the posterior cingulate cortex as well as precuneus and found strong correlations between the functional connectivity and outcome in comatose CA survivors. A structural study found widespread thinner cortex regions in patients with comatose CA that were associated with impaired memory performance (14). Another voxel-based morphometry study described gray matter atrophy after CA (15). They also showed that the reductions in the volume of several brain areas were significantly correlated with neuropsychological impairments (15). While a sizable percentage of neuroimaging studies have focused on comatose CA survivors (10–14), very little attention has been paid specifically to those survivors with preserved neurological function (16–18). We only found scarce structural studies which reported deficits in several brain regions concerning cognitive functions in the ‘good outcome’ survivors (16–18). However, to the best of our knowledge, no study has explored the functional brain alterations in the CA survivors with good neurological recovery.

Resting-state functional magnetic resonance imaging (rs-fMRI) is a noninvasive brain imaging technique for evaluating hemodynamic changes induced by neural activity (19). The amplitude of low-frequency fluctuation (ALFF) and regional homogeneity (ReHo) methods can be used to evaluate the regional intensity and synchronization of spontaneous fMRI signals and provide important information about the functional activity of the brain (20). Based on the findings of previous structural research, we hypothesized that we would find alterations in the brain areas related to cognitive functions in CA survivors with favorable neurological outcomes.

Our study aimed (1) to compare the functional brain activity differences between CA survivors and healthy controls (HCs) using ALFF and ReHo methods and (2) to examine the relationships between functional brain measures and clinical variables, especially cognitive performance, in the patient group.

## 2. Materials and methods

### 2.1. Subjects

This study was approved by the Ethics Committee of the First Affiliated Hospital of Nanjing Medical University. Informed consent was obtained from all subjects. Between May 2020 and May 2021, we recruited 16 patients with CA according to the following criteria: (1) documented cardiopulmonary resuscitation for spontaneous CA, (2) 18 years or older, and (3) score 1 or 2 on the Cerebral Performance Categories (CPC). Criteria for exclusion were: (1) Glasgow Coma Scale (GCS) score < 8, (2) pre-existing neurologic or psychiatric disorders, (3) history of traumatic brain injury and brain surgery, (4) contraindication of MRI examination, or (5) abnormal brain parenchyma on brain MRI. Finally, 13 patients were enrolled in our study (two had implanted cardioverter defibrillator and one had inadequate imaging quality). The control group consisted of 13 age- and sex-matched healthy volunteers with no history of neurologic or psychiatric disorders or brain injury. All the participants were right-handed.

### 2.2. Sample size calculation

Sample size was calculated according to the following formula:


$$n_{A}=\kappa n_{B}\;\mathrm{and}\;n_{B}=\left(1+\mathrm{1\kappa}\right){\left[\sigma \left(z1-\alpha /2+z1-\beta \right)/\left(\mu_{A}-\mu_{B}\right)\right]}^{2},$$

z=(μA−μB)/σ√(1/nA+1/nB)

Where μ is the mean value for each group, σ is the standard deviation, and α is the type I error and β is the type II error, meaning 1 − β is power. The α was set at 0.05 and 1 − β at 0.9 for calculation. According to the results of the preliminary experiment, the calculated threshold sample size for each group was 10.

### 2.3. Clinical assessment

Clinical data including time to return of spontaneous circulation (ROSC), CPC, modified Rankin Scale (mRS), etiology causes, and type of CA were collected. CPC is the predominant outcome measure for patients with CA and describes five levels of cerebral functioning (21). Patients with scores of 1 or 2 on CPC have normal or mild neurologic and psychological deficits but are independent in most activities of daily living. mRS is an outcome scale to check for recovery and the degree of continued disabilities (22). The scale runs from 0 to 6, running from perfect health without symptoms to death. Individuals with an mRS score of 0 had no symptoms, 1 had no significant physical disabilities, 2 had minor disabilities, and 3 had moderate disabilities. Besides that, the Montreal Cognitive Assessment (MoCA) was conducted for each participant. MoCA is a commonly used method for assessment of cognitive functions, including orientation, memory, visuospatial/executive functions, naming, registration, attention, and abstraction (23). Scores on the MocA range from 0 to 30. A score of 26 or higher is considered to be normal.

### 2.4. MRI data acquisition

MRI scanning was performed at an average of 14.85 ± 4.90 days after CA when the patient was in a stable condition, and the MRI-incompatible devices were no longer needed. The patients were monitored by an intensive care unit provider during transportation and scanning. All the patients and healthy participants were instructed to stay awake with their eyes closed and think about nothing during the scan.

All subjects were scanned in a 3.0 T MR scanner (MAGNETOM Skyra, Siemens Healthcare, Erlangen, Germany) with a 20-channel head and neck coil. A sagittal 3D high-resolution T1-weighted imaging was acquired with magnetization-prepared rapid gradient echo sequence. The parameters were as follows: repetition time (TR) = 1900 ms; echo time (TE) = 2.45 ms; voxel size =1 × 1 × 1 mm^3^; field of view (FOV) = 256 × 256 mm^2^; matrix size = 256 × 256; slice thickness = 1 mm; 176 slices. Functional images were obtained by an axial echo planar imaging sequence with the following parameters: TR = 2000 ms; TE = 30 ms; voxel size = 3.75 mm × 3.75 mm × 4 mm; FOV = 240 × 240 mm^2^; matrix size = 64 × 64; slice thickness = 4.0 mm; 35 slices. The scanning time was 12 min and 26 s in total. The images were promptly inspected for quality control after scanning. The subject was instructed to remain still and the procedure was repeated if the picture quality was subpar due to head movement.

### 2.5. Rs-fMRI data processing

A resting state fMRI data analysis toolkit (RESTplus, http://www.restfmri.net) based on Statistical Parametric Mapping software (SPM, http://www.fil.ion.ucl.ac.uk /spm) was used to conduct the rs-fMRI data preprocessing. Firstly, dicom data files were converted into nifti images and the first 10 time points were deleted. Then, slice timing and head motion correction were performed to make the remaining images slice-time-corrected and realigned to the first volume. Next, realigned images were spatially normalized to the Montreal Neurological Institute template (resampling voxel size = 3 mm × 3 mm × 3 mm). The images were then smoothed with a Gaussian kernel of 6 mm full-width at half-maximum (FWHM) and detrended to remove linear trends. Finally, the nuisance covariates, including head motion parameters as well as white matter and cerebrospinal fluid signals, were removed using linear regression. The head motion for each subject was checked, and subjects with head translation motion exceeding 3.0 mm or rotation over 3° would be discarded.

The analyses of ALFF and ReHo values were also conducted using the RESTplus toolkit. ALFF values were calculated with filtered signals within the low-frequency range without additional filtering. The fast Fourier transform algorithm was used to transform the time courses into a frequency domain. Then the average square root of the power spectrum across 0.01–0.08 Hz indicated the ALFF value. For ReHo calculation, filtering was conducted to collect the spatially standardized data in the frequency range of 0.01–0.08 Hz to reduce the influence of high-frequency noise. Then, ReHo was computed *via* Kendall’s Coefficient of Concordance as the regional coherence index of signal within the surrounding 27 voxels with smoothing at 6 mm FWHM Gaussian kernel.

### 2.6. Statistical analysis

The between-group comparison of ALFF and ReHo values was performed by the statistical module of Data Processing & Analysis of Brain Imaging (DPABI, http://www.rfmri.org/dpabi). Two-sample *t*-test was conducted to compare the differences in the ALFF and ReHo values between patients with CA and HCs with age, sex, and years of education as covariates. Statistical significance was determined by the Gaussian Random Field (GRF) correction with a threshold of two-tailed voxel-level *p* < 0.001 and cluster-level *p* < 0.05.

Statistical evaluations of demographic and clinical data were conducted in SPSS Version 25.0 (SPSS, Chicago, IL, United States). The assumption of normality was assessed with Kolmogorov–Smirnov test. Two-sample *t*-tests were adopted for comparisons of age and years of education between the two groups. Mann–Whitney *U* test was used to compare the MoCA scores. Fisher’s exact test was employed to analyze the sex ratio. All tests were two-tailed, and significance was set at *p* < 0.05.

The mean ALFF and ReHo values in each significant cluster were extracted for the following correlation analyses. Partial correlation analyses were performed to evaluate the relationships between the mean ALFF and ReHo values in significant clusters and clinical parameters after controlling for the effect of age, sex, and years of education. Multiple comparisons were corrected using the Bonferroni method (*p* < 0.05/4 = 0.0125).

## 3. Results

### 3.1. Demographic and clinical data

Tables 1, 2 present detailed demographic and clinical information. There were no significant differences in age (*p* = 0.713), sex (*p* > 0.999), and years of education (*p* = 0.895) between CA patients and HCs. The patient group showed significantly lower scores of MoCA than HCs (*p* = 0.030). The causes of CA included myocarditis (4 patients, 30.8%), acute myocardial infarction (4 patients, 30.8%), pulmonary embolism (3 patients, 23.1%), kounis syndrome (1 patient, 7.7%), and asphyxia (1 patient, 7.7%). Twelve (92.3%) patients had in-hospital CA, and one patient (7.7%) had out-of-hospital *CA.* Five patients had a history of hypertension and three patients had a history of diabetes. After CA, the mean time for the patients to ROSC was 33.77 ± 23.87 min. The mean score for CPC was 1.08 ± 0.23 and mRS was 1.08 ± 0.95.

**Table 1:** Demographic and clinical characteristics of the patient group.

Age, y	Sex	Education, y	Etiology of CA	Type of CA	Time to ROSC, min	MoCA	CPC	mRS
55	M	4	Myocarditis	IHCA	7	23	1	1
65	F	8	Myocarditis	IHCA	52	20	1	1
34	M	9	Kounis syndrome	IHCA	10	27	1	0
63	M	9	Pulmonary embolism	IHCA	43	25	1	1
78	M	6	Pulmonary embolism	IHCA	53	22	1	2
40	M	12	AMI	IHCA	20	29	1	1
56	F	16	AMI	IHCA	80	28	1	0
25	M	18	Myocarditis	IHCA	50	30	1	0
34	M	15	Myocarditis	IHCA	5	29	1	0
70	M	12	AMI	IHCA	7	26	1	2
67	M	5	Pulmonary embolism	IHCA	39	20	2	3
45	F	14	Asphyxia	IHCA	8	28	1	1
48	F	12	AMI	OHCA	26	27	1	2

CA, cardiac arrest; ROSC, return of spontaneous circulation; CPC, Cerebral Performance Categories; MRI, magnetic resonance imaging; MoCA, Montreal Cognitive Assessment; CPC, Cerebral Performance Categories; mRS, modified Rankin Scale; GCS, Glasgow Coma Scale; M, male; F, female; AMI, acute myocardial infarction; IHCA, in-hospital cardiac arrest; OHCA, out-of-hospital cardiac arrest.

**Table 2:** Comparison of demographic and clinical data between CA and HC group.

Sample characteristics	CA patients	HC	*p* value
	(*n* = 13)	(*n* = 13)	
Age (year)	52.31 ± 16.14	50.15 ± 13.25	0.713
Sex (female/male)	9/4	9/4	>0.999
Education (year)	10.77 ± 4.36	10.54 ± 4.47	0.895
MoCA	25.69 ± 3.43	28.28 ± 1.04	0.030

CA, cardiac arrest; HC, healthy control; MoCA, Montreal Cognitive Assessment.

### 3.2. ALFF and ReHo differences

**Figure 1:**
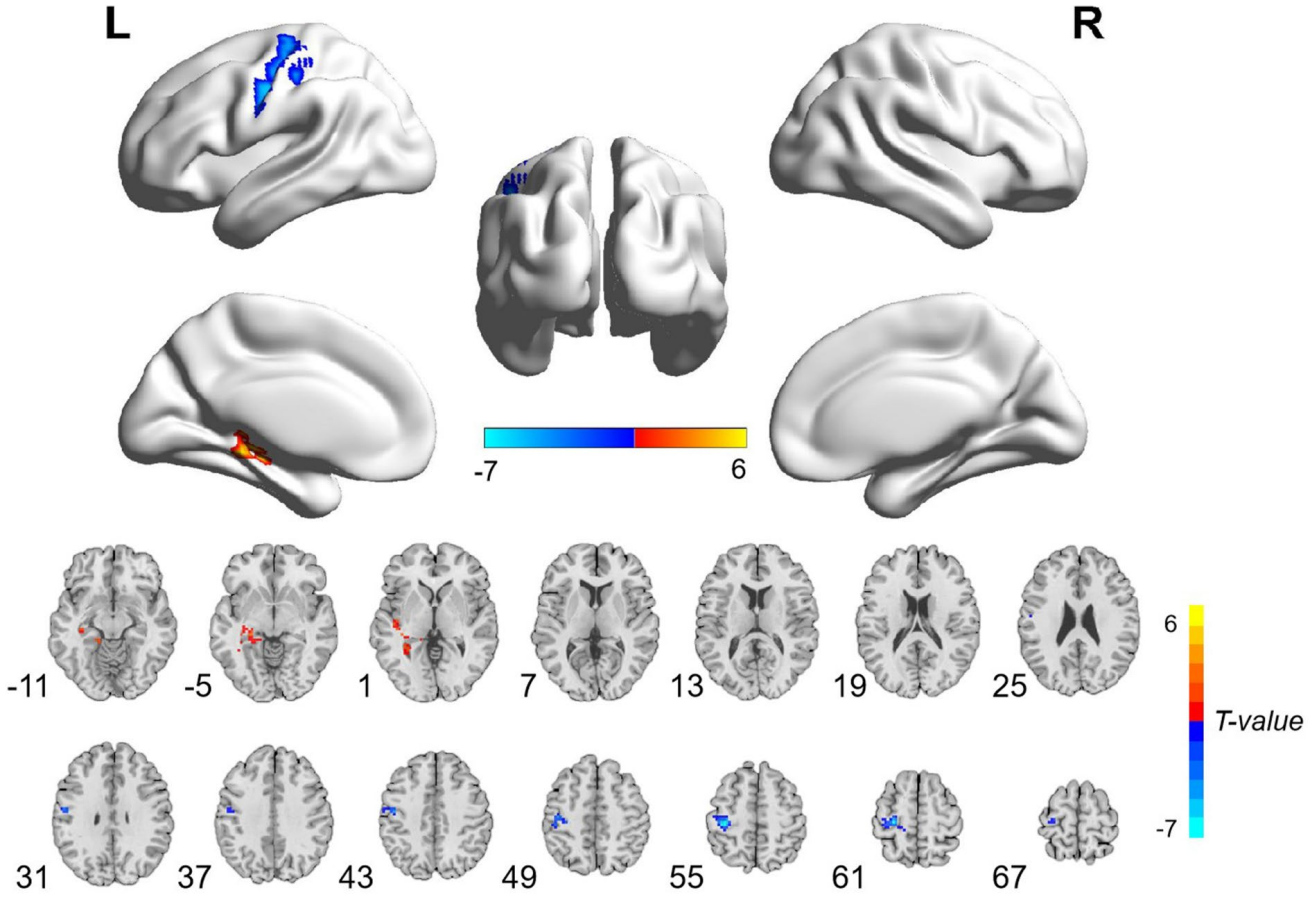
Brain regions with significant ALFF differences between patients with cardiac arrest and healthy controls. The patient group showed significantly decreased ALFF values in the left postcentral gyrus and precentral gyrus and increased ALFF values in the left hippocampus and parahippocampal gyrus (voxel-level *p* < 0.001 and cluster-level *p* < 0.05 with GRF correction). L, left; R, right; ALFF, amplitude of low frequency; GRF, Gaussian random field.

**Figure 2:**
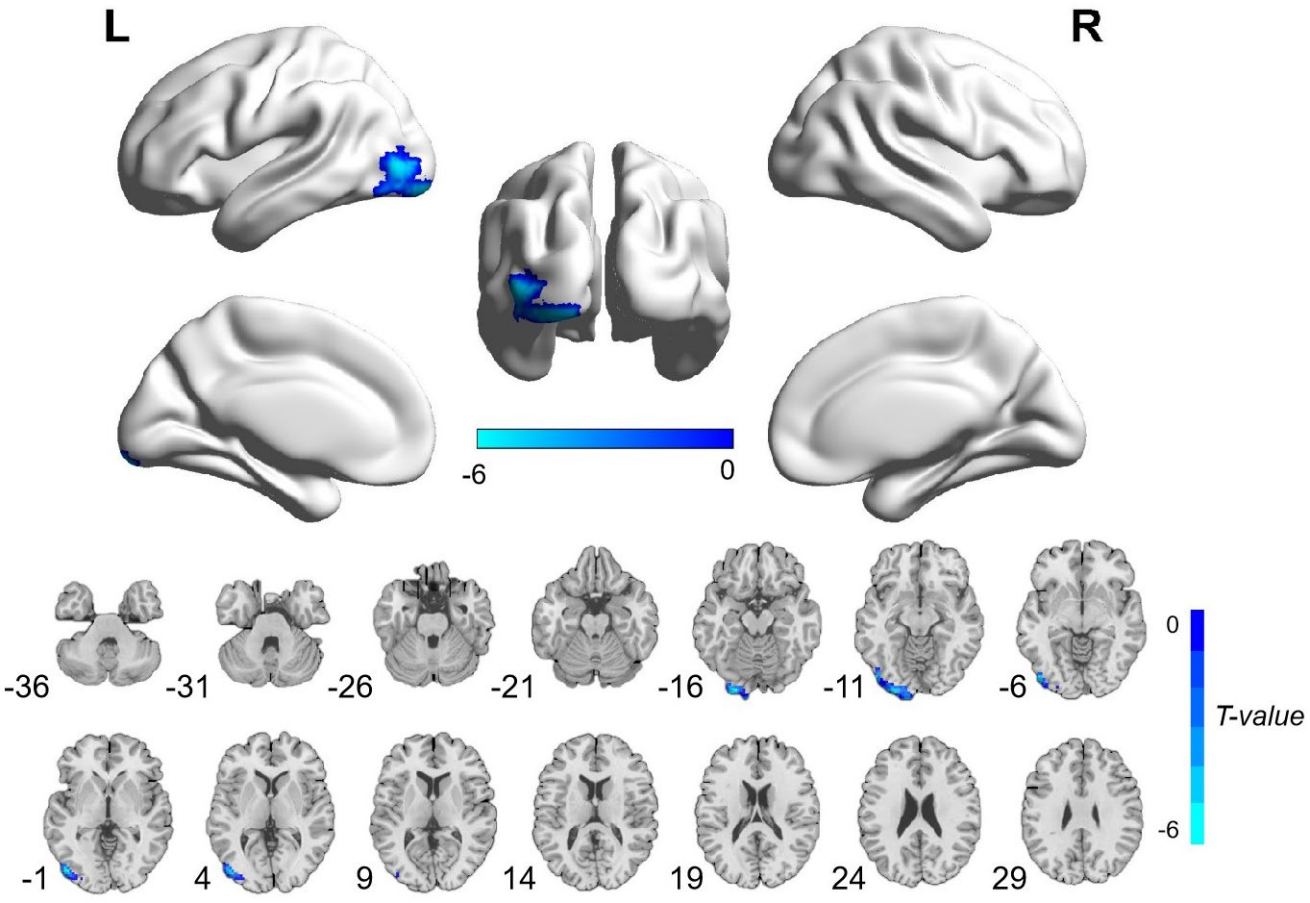
Brain regions with significant ReHo differences between patients with cardiac arrest and healthy controls. The difference primarily existed in the left inferior occipital gyrus and middle occipital gyrus (voxel-level *p* < 0.001 and cluster-level *p* < 0.05 with GRF correction). L, left; R, right; ReHo, regional homogeneity; GRF, Gaussian random field.

The patients with CA showed significantly decreased ALFF values in the left postcentral gyrus and precentral gyrus and increased ALFF values in the left hippocampus and parahippocampal gyrus than HCs (Table 3; Figure 1; voxel-level *p* < 0.001 and cluster-level *p* < 0.05 with GRF correction). Furthermore, significantly decreased ReHo values were observed in the left inferior occipital gyrus and middle occipital gyrus in the patients compared with HCs (Table 3; Figure 2; voxel-level *p* < 0.001 and cluster-level *p* < 0.05 with GRF correction).

**Table 3:** Brain regions with significantly different functional activity between patients and healthy controls (voxel-level *p* < 0.001 and cluster-level *p* < 0.05 with GRF correction).

Brain regions	Laterality	Peak MNI coordinates			Cluster size	*t*-value
		X	Y	Z		
*ALFF*						
Hippocampus/Parahippocampal gyrus	L	−18	−36	−9	101	5.1542
Postcentral gyrus/Precentral gyrus	L	−39	−27	54	176	−6.8969
*ReHo*						
Inferior occipital gyrus/Middle occipital gyrus	L	−48	−84	−9	229	−5.5617

GRF, Gaussian random field; ALFF, amplitude of low frequency fluctuation; ReHo, regional homogeneity; L, left; MNI, Montreal Neurologic Institute.

### 3.3. Correlation analysis

**Figure 3:**
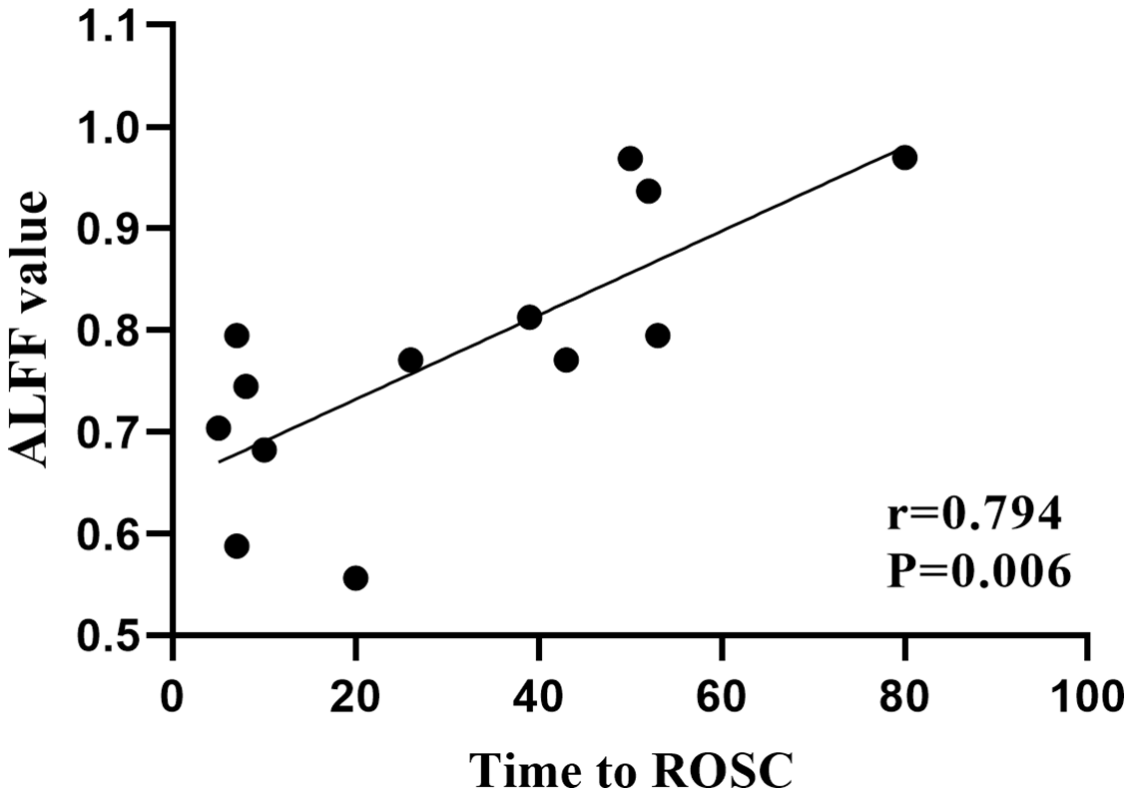
Significant correlations between clinical characteristics and ALFF values in patients with cardiac arrest. Mean ALFF values in the left hippocampus and parahippocampal gyrus were positively correlated with time to ROSC. ALFF, amplitude of low frequency; ROSC, return of spontaneous circulation.

In the patient group, mean ALFF values in the left hippocampus and parahippocampal gyrus were positively correlated with time to ROSC (*r* = 0.794, *p* = 0.006; Figure 3). No significant associations were found between ReHo values and clinical variables.

## 4. Discussion

Our study aimed to evaluate the functional brain activity alterations in the CA survivors with relatively good neurological outcomes using ALFF and ReHo methods. As a result, we found that CA patients exhibited significantly decreased ALFF values in the left postcentral gyrus and precentral gyrus and increased ALFF values in the left hippocampus and parahippocampal gyrus, as well as decreased ReHo values in the left inferior occipital gyrus and middle occipital gyrus. These results provide important information for understanding the neurological mechanism after *CA.*

The precentral and postcentral gyrus are key brain areas for motor and somatosensory functions, respectively (24, 25). Atrophy and deficits in the precentral gyrus have been documented in a number of movement-damaged disorders, including amyotrophic lateral sclerosis, stroke, and spinal cord injury, indicating its leading role in controlling body movement (26–29). Likewise, impairments in the postcentral gyrus were observed in several diseases with sensory disturbances, such as multiple sclerosis, hemiplegia, and autism spectrum disorders (30–32). As we know, motor and somatosensory deficits resulting from hypoxia-anoxia ischemic brain injury are common sequelae in patients with CA (33, 34). Although all of our patients achieved relatively good neurological outcomes, some of the patients still suffered from mild motorial and sensorial symptoms. Warenits et al. (35) found decreased neuronal function in the motor cortex, which is located in the precentral gyrus, and speculated it to be associated with motor deficits after CA in rats. Middleton et al. (36) discovered diminished brain function in the somatosensory cortex in developing rats after *CA.* Therefore, the decreased ALFF values in the precentral and postcentral gyrus may be associated with motor and somatosensory disabilities in patients with *CA.*

The occipital lobe is an integral part of the visual system and is involved in spatial processing (37). Visuospatial dysfunction is a rare but essential cognitive disease in the survivors of CA (34). Ørbo et al. (14) observed significantly thinner cortex in the left occipital lobe and correlated it with cognitive function alterations in patients with *CA.* In agreement with this finding, we also found significantly decreased functional activity in the left occipital lobe in patients with *CA.* Although the specific visuospatial examinations were not performed in the current study, we found that the overall cognitive measures were obviously reduced in patients with CA compared with HCs. Taken together, the decreased ReHo values in the left inferior occipital gyrus and middle occipital gyrus, which indicated decreased functional activity in visuo-spatial-related brain areas, may be associated with cognitive impairment in patients with *CA.*

The hippocampus is responsible for processing cognitive information, especially memory information (38, 39). The hippocampus and parahippocampal gyrus work as an entity to manage the procedures of memory encoding retrieval (40). Memory dysfunction, as the most common cognitive impairment in the survivors after CA, has been found to be related to the hippocampus deficit in this population (5, 6, 17, 18). Stamenova et al. (17) found reduced volumes in the bilateral hippocampus, which were correlated with verbal memory in patients with *CA.* Ørbo et al. (17) likewise detected the correlation between verbal memory and hippocampal subfield subiculum in patients with *CA.* In the present study, the average score of MoCA in the patient group was 25.69 ± 3.43, which was just beneath the normal line, indicating mild cognitive impairment (23). Thus, we suspected that the increased functional activity in the hippocampus and parahippocampal gyrus might reflect a compensatory mechanism of the brain to maintain a relatively desired function. A positive correlation was found between ALFF values in the hippocampus and parahippocampal gyrus and the time to ROSC, indicating that a longer anoxia time of the neurons would lead to more obviously increased functional activity in the above regions.

The results of this study provided neurological evidence for the clinical symptoms in patients with *CA.* Due to the mild to moderate nature of the cognitive dysfunction in the ‘good outcome’ CA survivors, most patients are discharged before the cognitive deficits are recognized (7, 8). Identifying patients at risk could be helpful for clinicians to provide comprehensive treatments for better rehabilitation. As an adjunct to clinical assessments, neuroimaging may be a promising technique for evaluating the cognitive status of patients with *CA.* Additional comparisons between functional and structural brain alterations would provide more insight into the neuropathological mechanism underlying the clinical symptoms in patients with *CA.* Longitudinal studies will also be helpful in elucidating the relationship between the development of symptoms and the changes in functional brain activity.

There are several limitations that should be noted within this study. Firstly, the sample size was relatively small, which is a common limitation of neuroimaging studies for CA survivors due to the low survival rate. Secondly, we excluded the CA survivors with implanted cardioverter defibrillators, which accounted for a fraction of those patients. Thus, our results might not be fully representative of the typical CA population. Thirdly, the cognition of our participants was assessed using MoCA, which is a brief cognitive screening test for mild cognitive impairments. Additional precise evaluations of CA patients’ most vulnerable cognitive functions (e.g., memory, attention, and executive functions) would expand the current findings. Finally, hypertension and diabetes, which are common diseases in the elderly, may have a certain impact on cognitive function. Future subgroup analysis would further enable us to get a more comprehensive understanding of the brain changes in patients with *CA.*

In conclusion, our results provided insight into the mechanisms underlying the cognitive impairments in CA survivors with favorable neurological outcome. The study may be helpful in prompting new research areas to extend these findings to diagnostic and therapeutic benefits.

## Data availability statement

The raw data supporting the conclusions of this article will be made available by the authors, without undue reservation.

## Ethics statement

The studies involving human participants were reviewed and approved by Ethics Committee of the First Affiliated Hospital of Nanjing Medical University. The patients/participants provided their written informed consent to participate in this study.

## Author contributions

QW: data curation, formal analysis, methodology, and writing original draft. G-NW: investigation, methodology, and writing original draft. HH: methodology and writing - review and editing. X-FC and X-QX: writing - review and editing. J-SZ and F-YW: conceptualization and writing - review and editing. All authors contributed to the article and approved the submitted version.

## Funding

Jiangsu Province Hospital (the First Affiliated Hospital with Nanjing Medical University) Clinical Capacity Enhancement Project JSPH-MC-2021-8 to X-QX.

## Conflict of interest

The authors declare that the research was conducted in the absence of any commercial or financial relationships that could be construed as a potential conflict of interest.

## Publisher’s note

All claims expressed in this article are solely those of the authors and do not necessarily represent those of their affiliated organizations, or those of the publisher, the editors and the reviewers. Any product that may be evaluated in this article, or claim that may be made by its manufacturer, is not guaranteed or endorsed by the publisher.
